# Ultrafast self-trapping of photoexcited carriers sets the upper limit on antimony trisulfide photovoltaic devices

**DOI:** 10.1038/s41467-019-12445-6

**Published:** 2019-10-04

**Authors:** Zhaoliang Yang, Xiaomin Wang, Yuzhong Chen, Zhenfa Zheng, Zeng Chen, Wenqi Xu, Weimin Liu, Yang (Michael) Yang, Jin Zhao, Tao Chen, Haiming Zhu

**Affiliations:** 1 Centre for Chemistry of High-Performance & Novel Materials, Department of Chemistry, Zhejiang University, Hangzhou, Zhejiang 310027 China; 2 CAS Key Laboratory of Materials for Energy Conversion, Department of Materials Science and Engineering, University of Science and Technology of China, Hefei, Anhui 230026 China; 3 Department of Physics, University of Science and Technology of China, Hefei, Anhui 230026 China; 4 School of Physical Science and Technology, ShanghaiTech University, Shanghai, 201210 China; 5 State Key Laboratory of Modern Optical Instrumentation, College of Optical Science and Engineering, Zhejiang University, Hangzhou, 310027 Zhejiang China

**Keywords:** Excited states, Solar cells

## Abstract

Antimony trisulfide (Sb_2_S_3_) is considered to be a promising photovoltaic material; however, the performance is yet to be satisfactory. Poor power conversion efficiency and large open circuit voltage loss have been usually ascribed to interface and bulk extrinsic defects By performing a spectroscopy study on Sb_2_S_3_ polycrystalline films and single crystal, we show commonly existed characteristics including redshifted photoluminescence with 0.6 eV Stokes shift, and a few picosecond carrier trapping without saturation at carrier density as high as approximately 10^20^ cm^−3^. These features, together with polarized trap emission from Sb_2_S_3_ single crystal, strongly suggest that photoexcited carriers in Sb_2_S_3_ are intrinsically self-trapped by lattice deformation, instead of by extrinsic defects. The proposed self-trapping explains spectroscopic results and rationalizes the large open circuit voltage loss and near-unity carrier collection efficiency in Sb_2_S_3_ thin film solar cells. Self-trapping sets the upper limit on maximum open circuit voltage (approximately 0.8 V) and thus power conversion efficiency (approximately 16 %) for Sb_2_S_3_ solar cells.

## Abstract (web summary)

Antimony trisulfide has a proper bandgap of 1.7 eV for making solar cells but the devices suffer from severe voltage loss. Here Yang et al. propose that the photoexcited carriers are self-trapped by lattice deformation, which places a thermodynamic limit of only 0.8 V for the open circuit voltage.

## Introduction

**Fig. 1:**
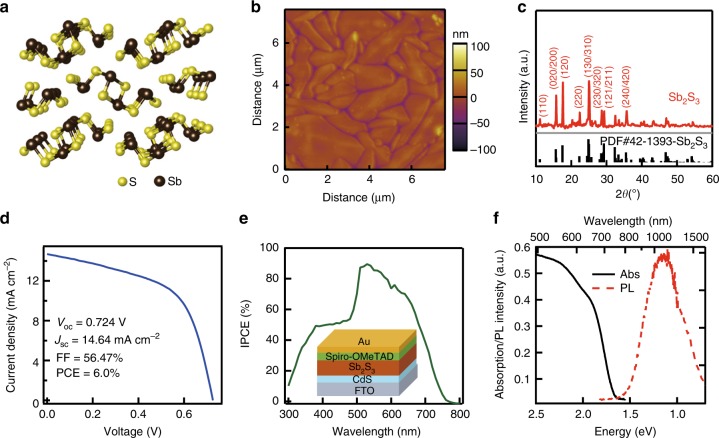
Characterization of hydrothermal grown Sb_2_S_3_ polycrystalline film. **a** Perspective review of Sb_2_S_3_ crystal structure projected on the [001] (or ab) plane. **b** AFM image and (**c**) XRD pattern of hydrothermal Sb_2_S_3_ polycrystalline film on CdS/FTO substrate. **d** *J–V* curve and (**e**) IPCE curve of a representative Sb_2_S_3_ solar cell. Inset: FTO/CdS/Sb_2_S_3_/Spiro-OMeTAD/Au solar cell device structure. **f** Absorption and PL spectra of hydrothermal Sb_2_S_3_ thin film

The exploration of semiconductor material for low-cost, stable, and efficient thin-film photovoltaics has been a key target for solar energy conversion. Among them, Cu(In,Ga)Se_2_, CdTe, and organic–inorganic hybrid perovskites play the leading role with power-conversion efficiencies (PCEs) above 20%, but on the other hand show various limitations imposed by element scarcity, stability, and environmental concerns. Recently, binary semiconductors antimony chalcogenides including Sb_2_S_3_ and Sb_2_Se_3_ emerge as promising materials due to their ideal bandgaps (*E*_g_ of 1.7 eV for Sb_2_S_3_ and 1.2 eV for Sb_2_Se_3_), large absorption cross-section, earth-abundance and environmental-friendly and stable characters^1–3^. They have fixed orthorhombic phase with infinite one-dimensional (1D) ribbons along the [001] (or c) direction (Fig. 1a for Sb_2_S_3_), avoiding complicated phase control during processing. In particular, Sb_2_S_3_ with *E*_g_ of 1.7 eV has been considered as a perfect component for the top subcell in Si-based tandem solar cells^2^.

Sb_2_S_3_ and Sb_2_Se_3_ solar cells of both sensitized and planar configurations have been extensively reported in recent years^4–16^. However, their solar cell performances remain unsatisfactory to date^2,3^. The record PCE is 7.5%^5^ and 9.2%^9^ for Sb_2_S_3_ and Sb_2_Se_3_ solar cells, respectively, much lower than that of CdTe or perovskite thin-film solar cells with similar bandgaps. While fill factor (FF) up to 70% and near-unity internal quantum efficiency have been achieved, the open-circuit voltage (*V*_oc_) is surprisingly low, regardless of fabrication and pre/post-treatment methods^1–3^. For example, using either chemical bath deposition, thermal evaporation, or atomic layer deposition, *V*_oc_ for Sb_2_S_3_ solar cell always falls into between 0.6 and 0.8 V with a record value of 0.77 V^17^, which is only half of theoretical thermodynamic limit (1.4 V for *E*_g_ equals to 1.7 eV) under AM1.5 irradiance^2^.

The large *V*_oc_ loss in Sb_2_S_3_ solar cell has been generally attributed to the surface/interface trap states and/or defects/impurities in bulk^18–22^. Various extrinsic defects have been invoked, including surface sulfide state, interfacial states at Sb_2_S_3_-electrode contact, antimony oxides formation, and sulfur vacancy^18–22^. These defect states are speculated to trap carries and accelerate their recombination, leading to low density of photoexcited carriers in semiconductors thus low *V*_oc_^2,18,20^. Previous transient absorption (TA) and time-resolved terahertz spectroscopy measurements on Sb_2_S_3_ (Sb_2_Se_3_) nanocrystals and polycrystalline films indeed observed picosecond carrier localization/trapping process^19,22,23^. In the face of nearly clamped *V*_oc_ loss after significant efforts on material optimization and device engineering, a critical question naturally arises on whether there is any intrinsic limitation on this semiconductor as photovoltaic material.

Here, we seek to answer this critical fundamental question based on spectroscopic study of excited-state carrier properties in Sb_2_S_3_. By comparing polycrystalline Sb_2_S_3_ film of three different growth methods and high-quality stoichiometric Sb_2_S_3_ single crystals, we observe strongly Stokes-shifted PL and ultrafast picosecond carrier-trapping process in all samples. Saturation of trapped carrier is not observed at carrier density as high as 10^20^ cm^3^, which is too large to be related to extrinsic impurities or defects. Together with polarized trap emission from single crystals, these results strongly suggest that photoexcited carriers are self-trapped by lattice distortion in Sb_2_S_3_. This intrinsic self-trapping explains well the 0.6 V *V*_oc_ loss and thermally activated carrier transport and ultimately sets the upper bound for the PCE in Sb_2_S_3_ and Sb_2_Se_3_ photovoltaic devices.

## Results

### Optical study of polycrystalline films

We first prepared Sb_2_S_3_ polycrystalline thin films via in situ hydrothermal growth on CdS/FTO substrate with CdS as electron transport layer in solar cell (see the Methods section for details)^10^. This method has been employed to grow high-quality Sb_2_S_3_ films for solar cells, and the thickness of Sb_2_S_3_ layer can be controlled by reaction time and temperature^10,24^. The atomic force microscopy (AFM) image shown in Fig. 1b indicates that as-grown Sb_2_S_3_ film is smooth and compact with large grain size. The crystal structure of Sb_2_S_3_ film was confirmed by X-ray diffraction (XRD) (Fig. 1c), and diffraction peaks at 15.7°, 17.6°, 25.0°, and 28.6° can be indexed to orthorhombic stibnite structure (JCPDS #42-1393). The photovoltaic properties of hydrothermal grown Sb_2_S_3_ film (thickness of ~300 nm) was examined by assembling FTO/CdS/Sb_2_S_3_/Spiro-OMeTAD/Au structured solar cell device (Fig. 1e inset). The *J*–*V* curve and incident photon-to-electron conversion efficiency (IPCE) curve of Sb_2_S_3_ thin-film solar cell under AM1.5 illumination are shown in Fig. 1d and e, respectively. The solar cell exhibits a *V*_oc_ of 0.72 V, *J*_sc_ of 14.6 mA cm^−2^, *FF* of 56.5%, and PCE of 6%, among the top values for planar Sb_2_S_3_ thin-film solar cell^2^. IPCE curve shows a peak value >80% at 510–580 nm, and drops in shorter wavelength due to CdS buffer layer and longer wavelength due to reduced absorption. All these characterizations indicate high-quality Sb_2_S_3_ thin film by hydrothermal growth for photovoltaic devices.

The absorption and photoluminescence (PL) spectra of Sb_2_S_3_ thin-film (thickness of 160 nm) are shown in Fig. 1f. The absorption spectrum exhibits an onset at ~1.65 eV (750 nm) and a weak absorption peak at ~1.9 eV (650 nm). The absorption onset agrees well with the onset of IPCE curve, corresponding to the bandgap of the Sb_2_S_3_ film^2,25^. According to literatures and our calculation (Supplementary Fig. 1), Sb_2_S_3_ is an indirect bandgap semiconductor with indirect gap of 1.7 eV (the precise value depends on sample conditions) and direct gap only slightly higher (80 meV)^2^. PL property of Sb_2_S_3_ has been barely reported. Strikingly, we observed a strongly red-shifted and broad PL peak at 1.15 eV under CW excitation (532 nm), albeit with weak intensity. This corresponds to a Stokes shift as large as ~500 meV, which can only be ascribed to emission from trap states rather than band-edge states.

**Fig. 2:**
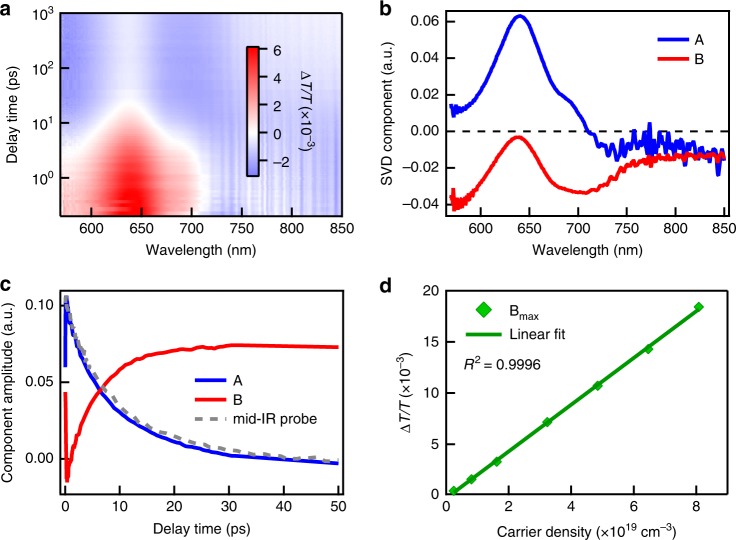
TA study of hydrothermal grown the Sb_2_S_3_ polycrystalline film. **a** 2D color plot of TA spectra of as-grown Sb_2_S_3_ polycrystalline film. **b** Principle spectral components and (**c**) associated kinetics from SVD analysis. Also shown in Fig. 2c is the mid-IR (5 μm) probe kinetics (gray-dashed line). **d** Maximum TA signal of B component (trapped carrier induced absorption) as a function of photoexcited carrier density and its linear fitting with *R*^2^ equal to 0.9996

To directly probe photoexcited carrier dynamics in a Sb_2_S_3_ polycrystalline film, we performed time-resolved TA measurements^19,25^. We excited and created carriers in Sb_2_S_3_ thin film with 2.1 eV pump pulse and after a certain delay time, probed it with either visible white light continuum or mid-IR probe pulse. While the visible photon mostly probes the interband electronic transitions, low-energy mid-IR photon (e.g., 5 μm) is dominated by Drude response of free carriers (Supplementary Fig. 2)^26^. Combining visible and mid-IR probe can provide a comprehensive picture about carrier dynamics in semiconductors. We note both free electron and hole contribute to TA signal, and their relative contribution is inversely proportional to their effective masses^26^. The 2D color plot of TA spectra of the Sb_2_S_3_ polycrystalline film is shown in Fig. 2a. We observed clear spectral evolution with increasing delay time, indicating the presence of multiple transient absorbing species and the conversion between them after photoexcitation. For TA results with spectral and temporal separated species, it is common to do analysis with sets of discrete time and wavelength. For a complex TA result, singular value decomposition (SVD) based on time–wavelength separability provides a facial and general method to describe TA result with the minimum number of transient species (base spectra) on a completely model-free basis^27^. The emergence and evolution of the species can be followed individually with time (base time traces). In order to disentangle transient species and characterize the photoexcitation dynamics in Sb_2_S_3_, we analyzed the TA data by the SVD method.

The TA data of Sb_2_S_3_ polycrystalline film can be well described by two principle components (A and B) with spectra and associated kinetics shown in Fig. 2b, c, respectively. The component A is dominated by bleach of optical transition with peak at 650 nm, which forms instantaneously after photoexcitation and decays in ps. The decay of component A closely follows the decay of mid-IR kinetics which directly probes free carrier population in the Sb_2_S_3_ film. Therefore, the component A can be assigned to photoexcited free carriers in Sb_2_S_3_ film which bleaches the ground-state transitions through both band filling and Columbic effect (e.g., screening, band renormalization)^26^. The other component B is dominated by induced absorption band with peaks at 700 nm and 565 nm. Interestingly, component B rises gradually in ps and its rising coincides with decay of A component/mid-IR kinetics, indicating that the decay of free carrier at band-edge leads to new transient species in the Sb_2_S_3_ polycrystalline film. Together with the trap emission, component B can be safely assigned to trapped carrier which provides a direct measure for trapped carrier population. Similar induced absorption feature has also been observed in photoexcited Sb_2_S_3_ nanocrystalline film and ascribed to sulfide radical (S^­•^)^19^. Based on these steady-state and time-resolved optical measurements, we can conclude that photoexcited free carriers (including both electrons and holes) in the Sb_2_S_3_ polycrystalline film get trapped in tens of ps, leading to trapped carrier-induced absorption in visible and strongly red-shifted near-IR PL. The detailed carrier-trapping kinetics will be analyzed later with a rate equation model.

To gain more hints about the nature of the trap states discussed above, we varied the excitation fluence thus the transient carrier density over a large range (1.5 × 10^18^ cm^−3^ to 8 × 10^19^ cm^−3^). If the trapping process in the Sb_2_S_3_ polycrystalline film are due to extrinsic defects, e.g., surface states, impurities, or atomic vacancies, the saturation of trapped carrier-induced absorption (component B) would be expected when the trap states are filled. We plotted the maximum amplitude of B component as a function of photoexcitation carrier density (Fig. 2d). Interestingly, we did not observe any saturation signature even at carrier density approaching 10^20^ cm^−3^. Such large trap density suggests carrier trapping in Sb_2_S_3_ is likely intrinsic.

### Optical study of single crystal

**Fig. 3:**
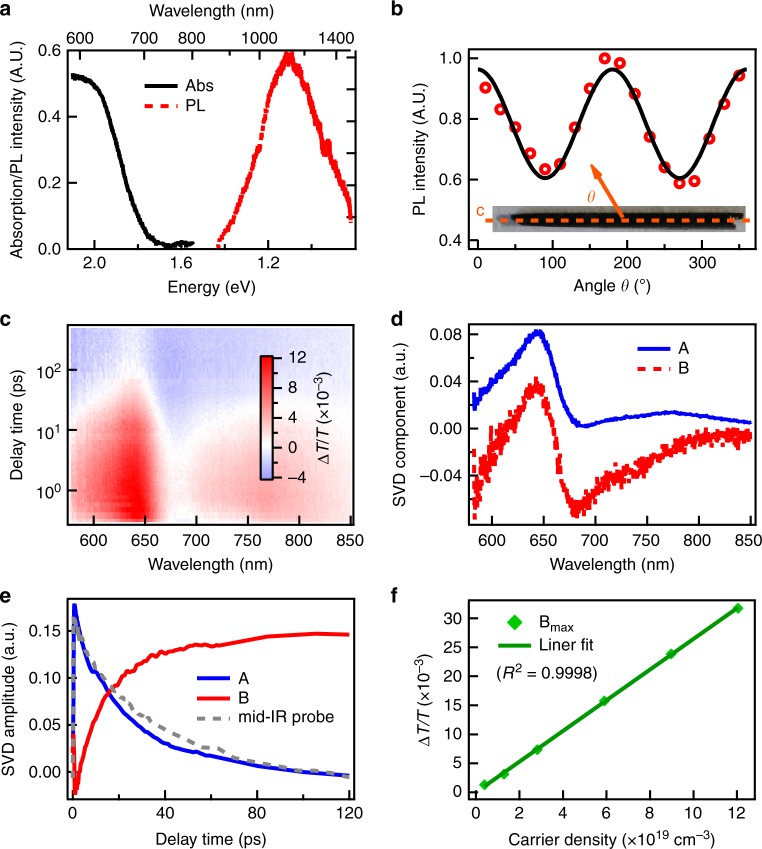
Steady state and TA study of Sb_2_S_3_ single crystal. **a** Absorption and PL spectra of Sb_2_S_3_ single crystal. **b** PL intensity from a Sb_2_S_3_ single crystal as a function of polarization detection angle (*θ*) and fitting with cos^2^*θ*. *θ* is defined to be 0 when detection polarization is along the crystal *c-*axis direction. Inset: optical image of a needle-like Sb_2_S_3_ single crystal. **c** 2D color plot of TA spectra of Sb_2_S_3_ single crystal. **d** Principle spectral components and (**e**) associated kinetics from SVD analysis. Also shown in Fig. 3e is the mid-IR (5 μm) probe kinetics (gray-dashed line). **f** Maximum TA signal of B component as a function of photoexcited carrier density and its linear fitting with *R*^2^ equal to 0.9998

To minimize possible deterioration by extrinsic defects and reveal the intrinsic photoexcited carrier properties in Sb_2_S_3_, we turn to zone-refined stoichiometric Sb_2_S_3_ single crystals grown by chemical vapor transport^28^. As shown in Fig. 3b inset, Sb_2_S_3_ single crystals have needle-like shape with length of a few cm (along *c*-axis direction) and width/height of ~1 mm, consistent with its quasi-1D crystal structure. Transmission X-Ray Laue photograph of Sb_2_S_3_ single crystal indicates high crystalline quality. We characterized the trap density of Sb_2_S_3_ single crystal by space charge-limited current method, which shows a very low trap density of 6.8 × 10^9^ cm^−3^ (Supplementary Fig. 3 and Supplementary Note 1). To probe the sample with transmitted light, we exfoliated the single crystal to an optically thin flake with thickness of 130 nm (Supplementary Fig. 4) for both steady state and TA measurements. The anisotropic crystal structure of Sb_2_S_3_ allows perfect cleavage perpendicular to the *b*-axis^2^.

The absorption spectrum of Sb_2_S_3_ single-crystal flake (Fig. 3a) exhibits an onset at 1.7 eV (730 nm) and a peak at 1.9 eV (650 nm), similar to that of the polycrystalline film. The PL of Sb_2_S_3_ single crystal under 532 nm CW excitation is also strongly red-shifted and broad with a peak at 1.1 eV, which represents substantial energy loss (0.6 eV) and can be assigned to trap emission. An important clue about trap state comes from examining trap emission polarization as a function of angle (*θ*) relative to the crystal *c*-axis (Fig. 3b). Surprisingly, the trap emission exhibits strong polarization along the *c-*axis, which can be fit by cos^2^*θ* function with a constant offset. The degree of polarization can be calculated by (*I*_∥_ − *I*_⊥_)/(*I*_∥_ + *I*_⊥_) to be 24%. This indicates transition associated with trapped hole and electron are preferentially orientated along the *c*-axis in Sb_2_S_3_ single crystal. Similar polarized near-IR PL was also observed from exfoliated Sb_2_S_3_ single crystal thin flakes, precluding the geometry effect as polarization origin. This polarized trap emission is unlikely due to surface states or impurities which usually would be randomly distributed and lead to isotropic trap emission.

More information about trap nature is provided by TA measurement on Sb_2_S_3_ single-crystal flake. The 2D plot of TA spectra is shown in Fig. 3c, which was analyzed by SVD method as above. The principle component spectra and associated kinetics are shown in Fig. 3d, e. Similar to the polycrystalline film, SVD analysis on Sb_2_S_3_ single-crystal flake yields a component A corresponding to photoexcited free carriers and a component B which is mainly contributed by induced absorption of trapped carriers. The main spectral features of principle components are similar in the Sb_2_S_3_ polycrystalline film and single-crystal flake with small variation likely due to inhomogeneous polycrystalline nature in former, justifying SVD method and suggesting their similar spectral origin. As shown in 3e, the decay of free carrier was confirmed with mid-IR probe and its decay process is also accompanied by the rise of trapped carrier-induced absorption. We varied the photoexcited carrier density (2 × 10^18^ cm^−3^ to 1.2 × 10^20^ cm^−3^) for Sb_2_S_3_ single-crystal flake and did not observe any saturation of trapped carrier induced absorption even when the carrier density was above 10^20^ cm^−3^ (Fig. 3f), which is too large to be related to extrinsic defects for Sb_2_S_3_ single crystal with a defect density of 6.8 × 10^9^ cm^−3^.

We performed similar steady state and transient optical measurements on the spin-coated and thermal-evaporated Sb_2_S_3_ polycrystalline films (see Supplementary Fig. 5 and Supplementary Fig. 6) and also observed a near-IR PL with ~0.6 eV Stokes shift and ultrafast carrier trapping to form similar induced absorption without any saturation. The close resemblance between Sb_2_S_3_ polycrystalline films of three different growth methods and Sb_2_S_3_ single crystal on carrier trapping properties, together with polarized trap emission in Sb_2_S_3_ single crystal, strongly suggest that ultrafast carrier trapping in Sb_2_S_3_ is associated with intrinsic self-trapping, instead of extrinsic defects as usually invoked.

## Discussion

**Fig. 4:**
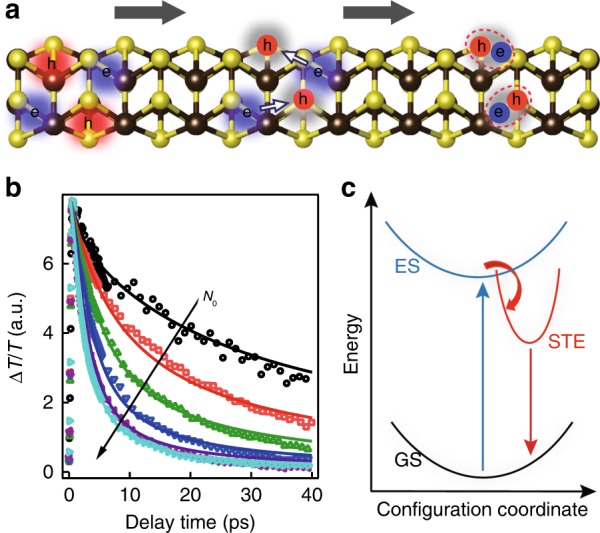
Self-trapping process. **a** Scheme showing two-step formation process of STEs in Sb_2_S_3_: hole is self-trapped first and then electron is captured by trapped hole to form STE. **b** Carrier-trapping kinetics (open symbols) under different photoexcited carrier densities *N*_0_ (5 × 10^18^ cm^−3^ to 1.2 × 10^20^ cm^−3^) for Sb_2_S_3_ single-crystal flake and their fits to two-step formation mechanism (solid lines). **c** Qualitative adiabatic potential energy curve showing photoexcitation from ground state (GS) to excited state (ES), which further evolves into STE state by deforming lattice and losing substantial energy

As was first introduced by Landau^29^, in a material with soft lattice and strong carrier–phonon coupling, free carriers (electrons or holes), or excitons (bound electron–hole pairs) can be trapped within potential wells produced by local lattice distortion, forming a quasiparticle called “polaron”^30^. When the short-range deformation–potential interaction is dominant over long range interaction, a small polaron forms as self-trapped carrier or exciton is localized within unit cell. Self-trapping as small polaron could occur even in a perfect crystal and create a transient defect state in bandgap by lattice deformation, leading to substantial energy loss and a Stokes-shifted PL (Fig. 4c).

Self-trapping is favored in materials with strong carrier–phonon interaction and small elastic constant, and has been observed in metal halide (e.g., NaCl), oxide (e.g., SiO_2_) and chalcogenide crystals (e.g., As_2_Se_3_)^31,32^. The elastic constant of Sb_2_S_3_ has been calculated^33^, and the value (~40) is as small as that of SiO_2_ and NaCl crystals^32^. The Huang-Rhys parameter of Sb_2_S_3_, which reflects carrier–phonon interaction, was estimated to be 38.5, which is as large as that of NaCl and Cs_2_AgInCl_6_^34^ where STE has been demonstrated (Supplementary Note 2). Dimensionality also plays an important role in self-trapping. Compared with three-dimensional system, free carrier in (quasi) 1D system has been predicted to be intrinsically unstable and tends to be self-trapped without barrier^31^. As it happens, Sb_2_S_3_ has a quasi-1D crystal structure composed with (S_4_S_6_)_n_ ribbons stacked with van der Waals interaction. Therefore, carrier/exciton self-trapping is very likely in Sb_2_S_3_ and can explain all the optical properties and photoexcitation dynamics we observed, including strongly red-shifted trap emission, picosecond carrier trapping dynamics, trapped carrier induced absorption without saturation at 10^20^ cm^−3^ carrier density, and polarized trap emission in single crystal. Similar spectroscopic characteristics have been observed in seemingly disparate materials where self-trapped carriers or excitons have been commonly observed^31^.

Self-trapped carriers also differ strikingly from free carriers in transport properties. Because of strong localization to a single site, the former moves incoherently through thermally activated hopping process which moves faster with temperature while the latter moves through coherent band-like transport with inversely temperature relationship^30,31^. Temperature-dependent electrical measurements have been performed on Sb_2_S_3_ polycrystalline films and single crystals^35–37^. A combined electric and magnetic measurement on Sb_2_S_3_ crystal show thermal-assisted hopping transport mechanism^35^, which can be well explained by carrier self-trapping in Sb_2_S_3_.

For free carriers, hole self-trapping instead of electron is generally observed in metal halides (e.g., AgCl, NaCl) and chalcogenides (e.g., As_2_S_3_, As_2_Se_3_) because of rich *p* orbital electrons in valance band, possible bond alternation, and small valance band width^31,32,38–40^. In additional to sulfide *p* electrons, Sb_2_S_3_ possess Sb 5 *s*^2^ inert lone-pair electrons with complex Sb–S chemical bonds and coordinations^2,41^. Electronic structure calculation also shows narrower valance band width than conduction band in Sb_2_S_3_ (Supplementary Fig. 1). All these suggest hole is more likely to be self-trapped than electron in Sb_2_S_3_. This is consistent with smaller hole mobility^21^ than electron^17^ and previously suggested accepter-like trap state in Sb_2_S_3_ crystal^35^.

Self-trapping of electrons and holes after photoexcitation eventually leads to self-trapped excitons (STEs)^31^. Based on Stokes shift, the trapping depth of STE is ~0.6 eV away from band-edge state. A temperature dependent study (between 77 K and 297 K) on STE emission from Sb_2_S_3_ single crystal shows negligible band-edge exciton emission and no change of STE emission intensity as a function temperature, which indicates small energy barrier (less than kT of ~6.6 meV for 77 K) from band-edge carrier to STE and large (>0.4 eV) trapping depth for STE based on a thermal quenching model simulation (see Supplementary Fig. 7 and Supplementary Note 3). STE can form through either direct exciton trapping of same photoexcited electron–hole pair or through a two-step mechanism: hole self-trapping occurs first through mono-molecular process and then electron is captured by self-trapped hole (STH) to form STE through bimolecular process (Fig. 4a). The underlying physics in two-step mechanism is that photoexcitation generates free electrons and holes and electrons can move away from their geminate holes and sample a certain volume before being captured by STH. These two mechanisms can be differentiated by examining the trapping process as a function of photoexcited carrier density^32^. Trapping process does not depend on density in first case, but increase in second one as electron-STH bimolecular trapping would be proportional to the number of pre-formed STH and electron. As shown in Fig. 4b for Sb_2_S_3_ single-crystal flake, free carrier-trapping kinetics (obtained from visible TA measurement) decay faster with increasing photoexcited carrier density (same density value as in Fig. 3f) and approaches saturation. The density-dependent decay kinetics can be well replicated by a two-step rate equation model with an intrinsic hole-trapping rate constant (***k***_HT_) and a bimolecular electron-STH capturing rate constant (Supplementary Note 4). We note in this model, we assume these two rate constants are independent on photoexcitation density in the investigated regime. The decay kinetics at high carrier density is limited by initial hole self-trapping step, thus approaches saturation. Fitting to the experimental kinetics (Fig. 4b) yields a hole intrinsic self-trapping lifetime (**1/***k*_HT_) of 1.8 ps and unambiguously demonstrates two-step trapping process for STE formation in Sb_2_S_3_ (Fig. 4a). In view of the large energy dissipated, it is likely bond alternation is involved for STE formation in Sb_2_S_3_. A combined study of advanced spectroscopic techniques (e.g., transient extreme-ultraviolet^42^ or X-ray absorption^43^) and theoretical calculations is required to identify local chemical and structural information of STE.

STE in the Sb_2_S_3_ polycrystalline film has a half-life time of 23 ns (Supplementary Fig. 8), persisting through the decay of TA signal. With reported photoexcitation diffusion coefficient of 6.8 × 10^−2^ cm^2 ^s^−1^ in the Sb_2_S_3_ polycrystalline film^21^, this corresponds to a diffusion length of 400 nm. We note this diffusion coefficient value is likely a mixture of free carrier and STE^23^ therefore this diffusion length should be considered as an upper bound for STE. This explains near-unity carrier collection efficiency in a Sb_2_S_3_ thin film solar cell, despite the formation of STE^8,10^. Since self-trapping is intrinsic to the crystal, equivalent sites occur in each unit cell, which allows thermal-assisted hopping transport. Indeed, the STE half-life time is significant shortened to be 6.9 ns on CdS/FTO substrate, suggesting efficient carrier extraction by transport in bulk followed by interfacial electron transfer to CdS (Supplementary Fig. 8). We also note there is band bending and build-in electric field in CdS/Sb_2_S_3_ junction, which can facilitate the dissociation of STE and carrier drift transport^10,14^.

Similar to extrinsic defect states, self-trapping localizes carriers to specific sites and creates defect states in the gap (Fig. 4c). While in general extrinsic defects undeniably affect excited state carrier lifetime and solar cell performance, self-trapping is primarily responsible for ultrafast carrier trapping and energy loss process in Sb_2_S_3_, instead of surface/interface/bulk extrinsic trap states usually assumed. Based on PL Stokes shift, self-trapping causes 0.5–0.6 eV energy loss, which well explains the near-clamped *V*_oc_ loss (0.63 V) between record *V*_oc_ value and theoretical value. Different from extrinsic trap states, self-trapping will exist even in perfect Sb_2_S_3_ crystal, setting the upper limit on *V*_oc_ and PCE of Sb_2_S_3_ solar cell. Assuming thermodynamic limit values of *J*_sc_ and FF remain same in Sb_2_S_3_, but *V*_oc_ is reduced from 1.4 V to 0.8 V due to self-trapping energy loss, maximum PCE of Sb_2_S_3_ solar cell will be 16% instead of 28.6%^2^. Although our studies here are all based on planer Sb_2_S_3_ thin films, carrier/exciton self-trapping should also occur in Sb_2_S_3_-sensitized photovoltaic devices with several nanometer thickness because of its intrinsic nature. There both extrinsic surface trapping and intrinsic self-trapping would affect excited-state carrier properties and device performances due to large surface area^5^. This study here calls for reconsideration of Sb_2_S_3_ and Sb_2_Se_3_ as the photovoltaic materials and designing and optimizing their optoelectronic devices.

## Methods

### Sample preparation

The Sb_2_S_3_ polycrystalline film on the CdS/FTO substrate was grown via hydrothermal method following our previous report^10^. Sb_2_S_3_ polycrystalline film on glass was prepared with spin-coating antimony-complex precursor solution and post annealing^13^. Thermal-evaporated Sb_2_S_3_ thin film was deposited on glass at a high vacuum (~10^−4^ Pa) and annealed on preheated hot plate at 300 °C for 2 min in a glove box. A Sb_2_S_3_ single crystal was grown by chemical vapor transport (Shanghai Onway Technology Co, Ltd)^28^.

### Device fabrication and measurement

The hole-transporting layer was prepared by spin-coating Spiro-OMeTAD chlorobenzene solution with the concentration of 36 mg mL^−1^ at 3000 rpm for 30 s and then with a post treatment at 100 °C for 10 min. Au counter electrode about 70 nm was deposited by a thermal evaporator under a pressure of 5.0 × 10^−4^ Pa. Current−voltage measurements of Sb_2_S_3_ solar cell was performed in a standard xenon-lamp-based solar simulator (Oriel Sol 3A, USA). The test was under a 100 mW cm^−2^ solar irradiation at room temperature. The solar simulator illumination intensity was calibrated by a monocrystalline silicon reference cell calibrated by the National Renewable Energy Laboratory (NREL). The external quantum efficiency (EQE, Model SPIEQ200) was measured using a single-source illumination system (halogen lamp) combined with a monochromator.

### Electronic structure calculation

The calculations were carried out using Vienna ab initio simulation package (VASP) with a plane wave basis set. The electron–nuclei interaction was described by the projector augmented wave (PAW) method. For the exchange-correlation functional, we used the generalized gradient approximation of Perdew–Burke–Ernzerhof (GGA-PBE). Structures were fully relaxed until residual forces on constituent atoms become smaller than 0.01 eV Å^−1^, and total electronic energies were converged to 10^−5^ eV. An energy cutoff parameter of 450 eV and a Monkhorst-Pack k-point sampling grid of 5 × 5 × 15 for unit cell and 3 × 3 × 8 for supercell were sufficient for convergence. We took into account the van der Waals interaction using a DFT-D2 approach (a nonlocal correction functional was added to account for dispersion interactions).

### Optical measurement

Absorption spectra of films were measured on a Cary 5000 UV-Vis-NIR absorption spectrometer with integrating sphere. Absorption of exfoliated flakes and room temperature photoluminescence (PL) were performed on a home-built microscope setup. We used a 532 -nm CW laser as excitation and collected the PL and sent to an EMCCD (ProEM: 16002, Princeton Instrument) for visible region and a liquid nitrogen cooled InGaAs detector (PyLon IR, Princeton Instrument) for near-IR region. For femtosecond TA measurements, the fundamental beam from Yb: KGW laser (Pharos, Light Conversion Ltd.) was separated to multiple paths and sent to ultrafast spectrometer (TA100, Time-Tech Spectra, LLC). One was introduced into a noncollinear optical parametric amplifier to generate pump pulse at a certain wavelength. Another path was focused onto a YAG crystal to produce white light continuum (520–950 nm) as probe light. The third one was introduced to a collinear optical parametric amplifier for mid-IR probe generation. Nanosecond TA measurements were carried out on ns spectrometer (ns-TA100, Time-Tech Spectra, LLC) with a white light supercontinuum laser as probe pulse. The transmitted probe light with (*T*_pump_) and without (*T*_unpump_) pump were collected and the normalized transmittance change Δ*T*/*T* was calculated by Δ*T*/*T* = (*T*_pump_ − *T*_unpump_)/*T*_unpump_.

## Supplementary information

Supplementary Information

Peer Review File

## Data Availability

The source data necessary to support the findings of this paper are available from the corresponding author upon request.
