# Supplementary material for: Ultrafast self-trapping of photoexcited carriers sets the upper limit on antimony trisulfide photovoltaic devices
Source: Nat Commun. 2019 Oct 4;10:4540. doi: 10.1038/s41467-019-12445-6 (PMC6778121; doi:10.1038/s41467-019-12445-6)
Supplement: Supplementary file 1 — Supplementary Information [file 41467_2019_12445_MOESM1_ESM.pdf]

Supplementary Information

Ultrafast Self-trapping of Photoexcited Carriers Sets the Upper Limit on Antimony Trisulfide Photovoltaic Devices

Yang et al.

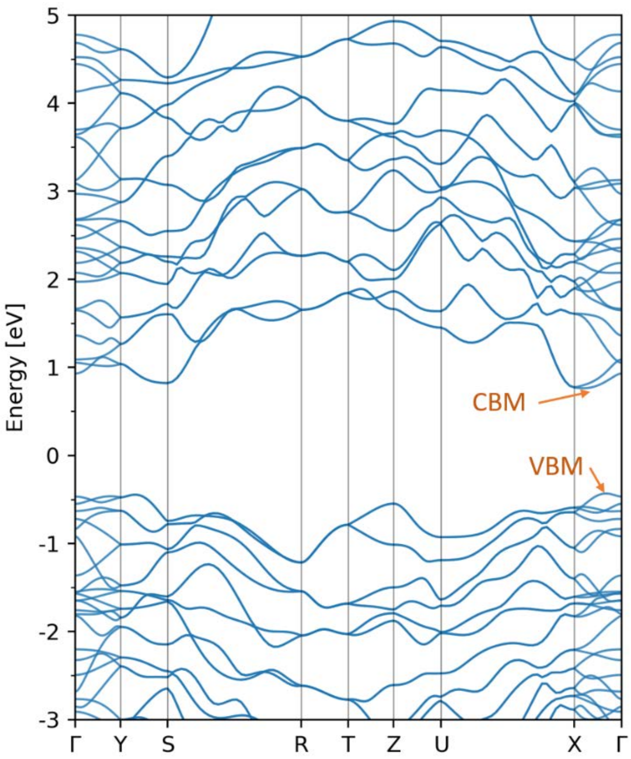

Supplementary Figure 1. Calculated electronic structure of Sb_2S_3, showing indirect bandgap nature. The lowest energy direct transition is ~ 80 meV high than indirect one.

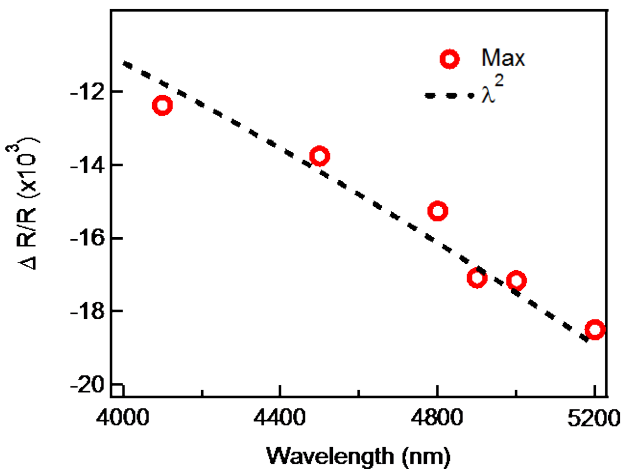

Supplementary Figure 2. Maximum mid-IR probe signal as a function of probe wavelength. The mid-IR response can be well described by Drude response of free carriers ($\propto \lambda^2$).

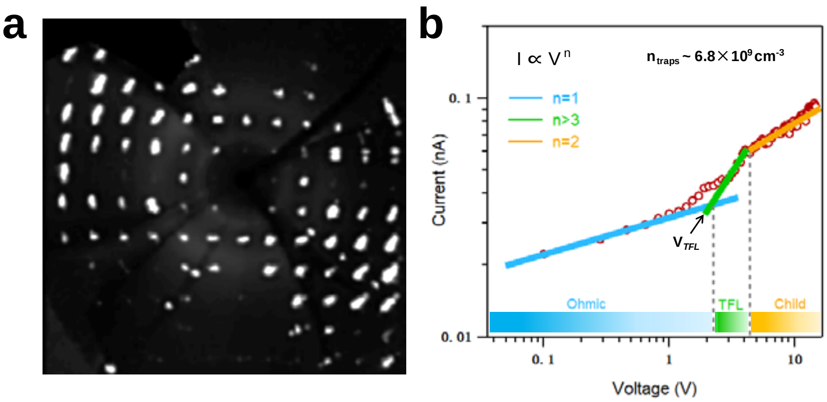

Supplementary Figure 3. (a) Transmission X-Ray Laue photograph of the needle-shaped Sb_2S_3 single crystal (b) I–V curves of Sb_2S_3 single crystal fitted by the space charge-limited current (SCLC) model measured, including three distinct regions: an Ohmic region(n=1), a trap filling limited (TFL) region(n>3) and a Child region(n=2)

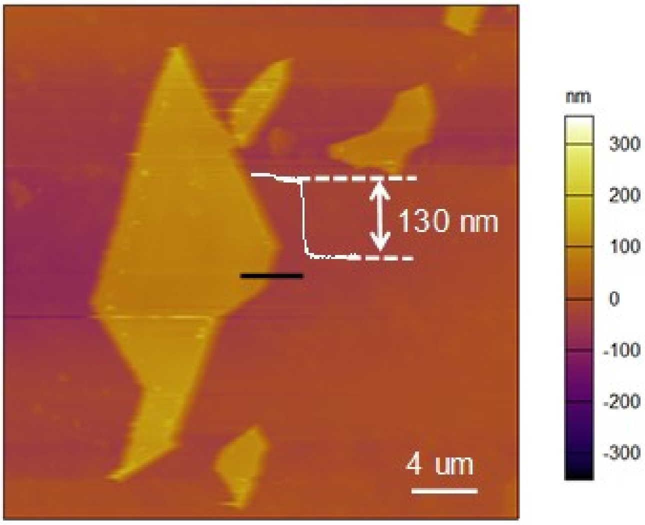

Supplementary Figure 4. AFM image of Sb2S3 single crystal flake with a thickness of ~ 130 nm.

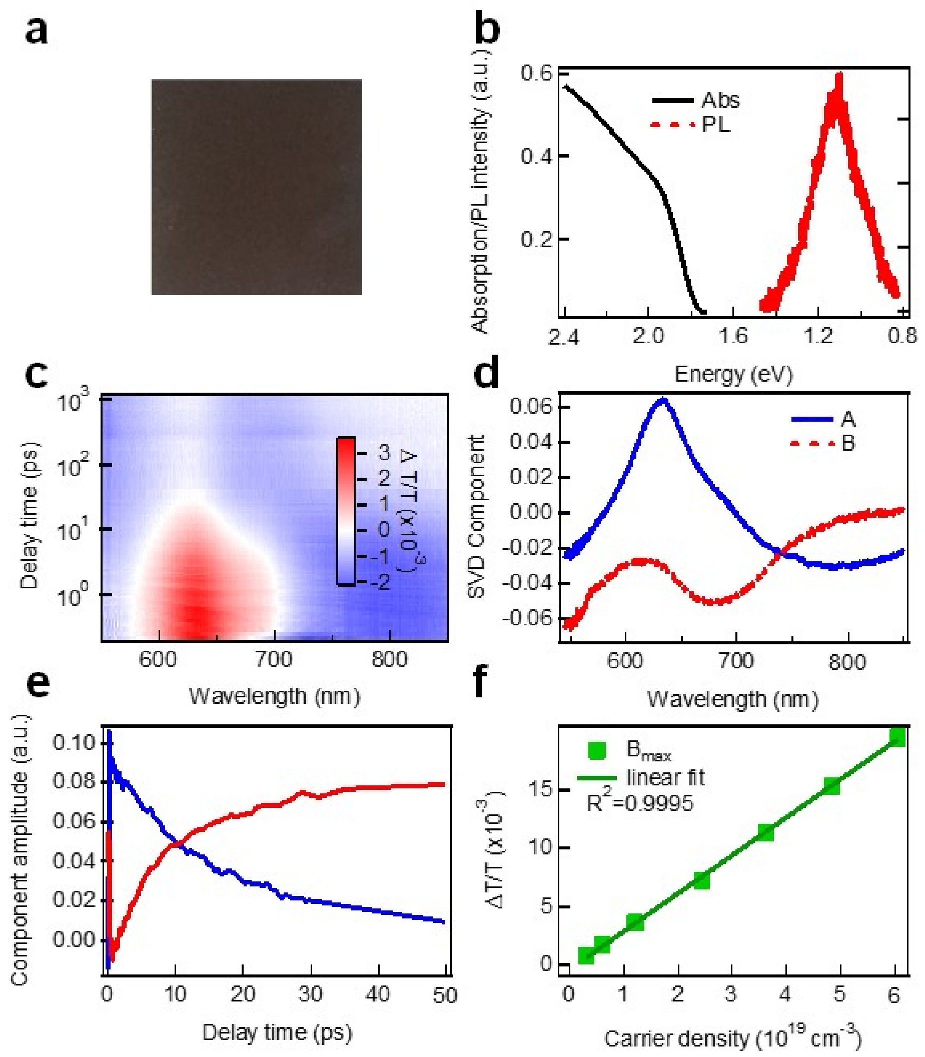

Supplementary Figure 5. (a) Optical image of spin coated Sb_2S_3 thin film on glass. Sample size: 1cm*1cm. (b) Absorption and PL spectra of Sb_2S_3 thin film. (c) 2D color plot of TA spectra of Sb_2S_3 thin film. (d) Principle spectral components and (e) associated kinetics from SVD analysis. (f) Maximum TA signal of B component as a function of photoexcited carrier density and its linear fitting.

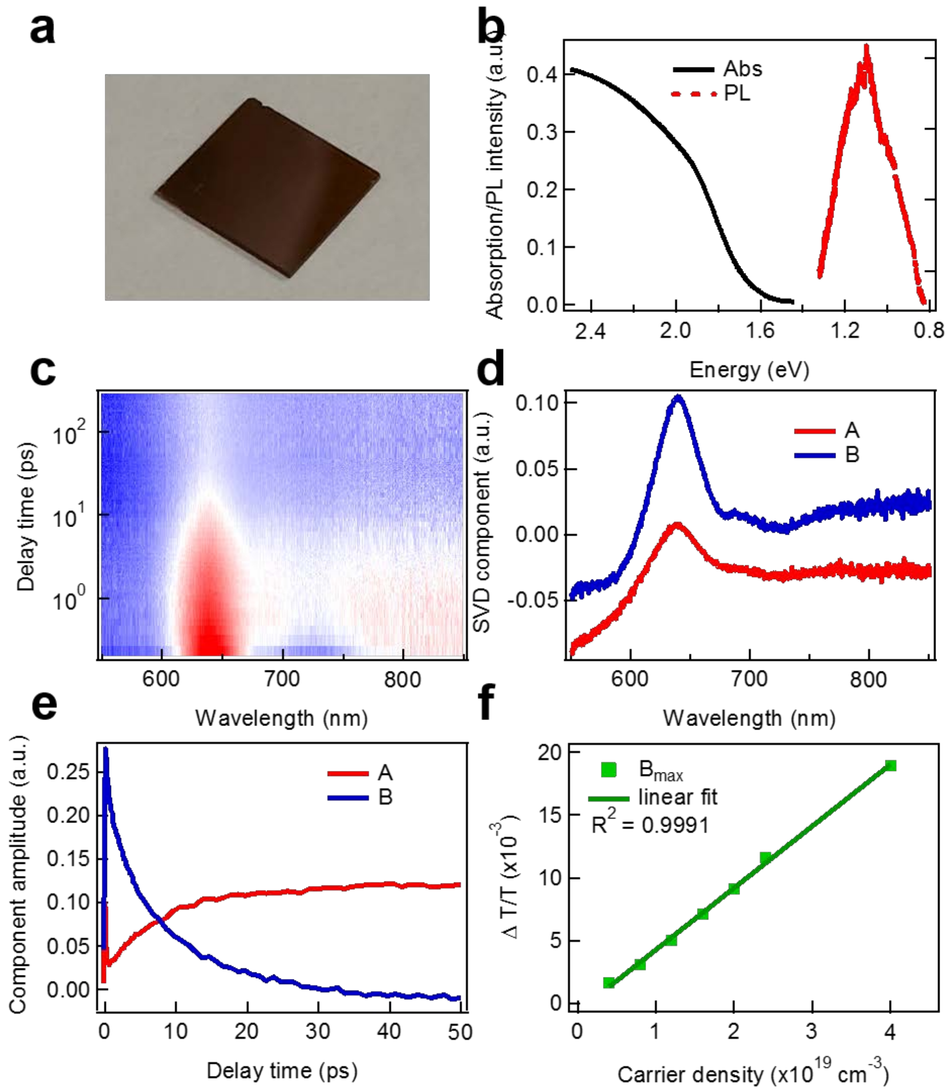

Supplementary Figure 6. (a) Optical image of thermal-evaporated Sb_2S_3 thin film on glass. Sample size: 1cm*1cm. (b) Absorption and PL spectra of Sb_2S_3 thin film. (c) 2D color plot of TA spectra of Sb_2S_3 thin film. (d) Principle spectral components and (e) associated kinetics from SVD analysis. (f) Maximum TA signal of B component as a function of photoexcited carrier density and its linear fitting.

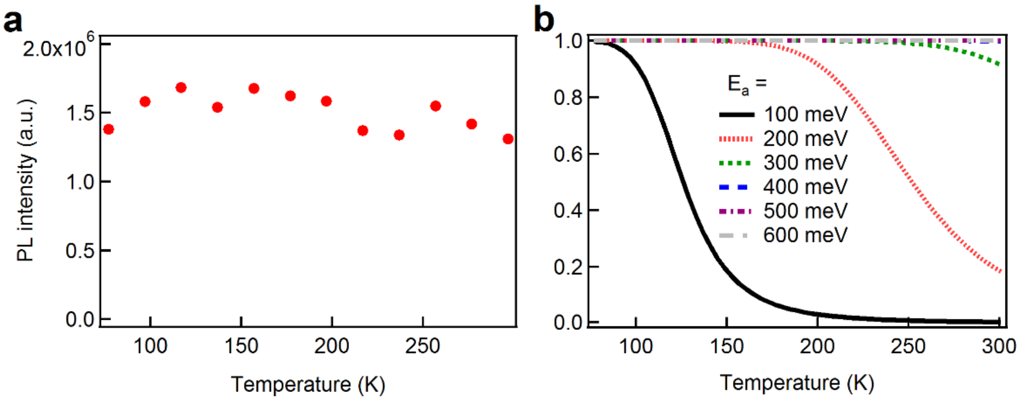

Supplementary Figure 7. (a) Integrated PL intensity of Sb_2S_3 single crystal flake as a function of temperature (b) Simulated temperature dependent PL intensity using a thermal quenching model with different activation energy.

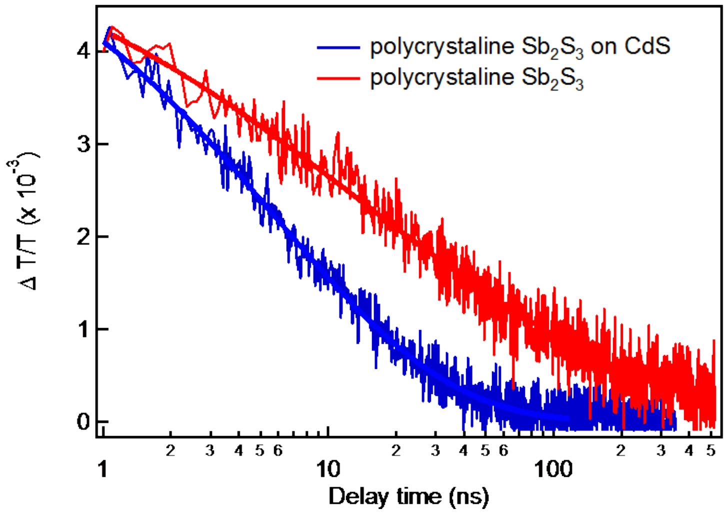

Supplementary Figure 8. The decay of STE in nanosecond time scale. STE lifetime is much shorter on CdS, suggesting efficient electron extraction.

Supplementary Note 1. Characterization of single crystal quality

The Transmission X-Ray Laue photograph of the needle shaped Sb_2S_3 single crystal is shown in Fig. S3a. It reveals that the obtained sample was a single crystal owing to the presence of Laue cones. In addition, the calculation result of lattice parameters a = 11.26, b = 11.33, and c = 3.85 Å with high indexing rate (92.1%, 94.7% and 89.5%, respectively), indicates high crystalline quality.

We employed the space charge-limited current (SCLC) with device structure of $Ag/Sb_2S_3/Ag$ to calculate the trap state density through the following equation:[1]

$$N_t = \frac{2\varepsilon_0 \varepsilon_r V_{TFL}}{qL^2} \quad (1)$$

where ε_0 is the vacuum permittivity, ε_r (= 7) is the relative dielectric constant of the Sb_2S_3, V_{TFL} is the onset voltage of trap filling limited (TFL) region (see Fig. S3b), q is the elemental charge and L is the distance between two electrodes (0.5 cm). According to the equation, trap density of Sb_2S_3 single crystal was calculated to be 6.8×10^9 cm^{-3}, which is a very low value. As a comparison, the trap density in $MAPbI_3$ single crystal was calculated to be $3\times10^{10} \sim 3\times10^{11}$ cm^{-3}.[2]

Supplementary Note 2. Estimate Huang-Rhys parameter based on Stokes shift

The coupling between photoexcitation and lattice phonons leads to the broad and Stokes shifted PL. Therefore, we can estimate the Huang-Rhys parameter S, which describes carrier phonon coupling, based on the Stokes shift E_{Stokes} by[3]

$$E_{Stokes} = (2S - 1)\hbar\omega \quad (2)$$

Based on the absorption spectrum and PL, E_{Stokes} ~ 600 meV. Previous coherent phonon measurement on Sb_2S_3 indicates a longitudinal optical phonon mode with a frequency of 63.74 cm-1 (~ 7.9 mV) couples to photoexcitation strongly. Based on these values, the Huang-Rhys parameter S was calculated to be 38.5, which is much larger than CdSe (1),[4] ZnSe (0.31),[5] $CsPbBr_3$ (3.22)[6] but similar to $Cs_2AgInCl_6$ (38.7)[7] and NaCl (30)[8] where STE have been demonstrated.

Supplementary Note 3. Temperature dependent PL intensity of Sb_2S_3 single crystal flake and thermal quenching modeling

To further confirm deeply trapped STE, we performed a temperature dependent PL measurement on Sb_2S_3 single crystal flake. The integrated PL intensity as a function of temperature is plotted in Fig. S6a, which shows negligible temperature dependence. We simulate the temperature dependent PL with a thermal quenching model assuming an upper unbound non-emissive state and the lower bound emissive state.[9, 10] In Sb_2S_3, STE PL comes only from lower bound state and thermal activation of STE with an activation/ionization energy E_a to band edge state leads to quenching of STE PL. Under CW excitation condition, STE PL intensity I should be

$$I(T) \propto \frac{1}{1+\left(\tau/\tau_0\right)e^{-E_a/kT}} \quad (3)$$

where τ is the lifetime of STE and τ_0 is the effect scattering time for thermally activated STE to upper band edge state. The latter is usually much faster than former and we choose $\tau/\tau_0 = 10^4$ in our simulation (this number does not affect the trend).

The simulated temperature dependent PL intensity for different E_a is shown in Fig. S6b. Comparing experimental and simulated results indicates E_as for STE is larger than 400 meV, which is constant with ~ 0.6 eV Stokes shift.

Supplementary Note 4. Rate equation model for two-step carrier trapping process

In the two-step carrier trapping process,[11] we denote for hole, N_{TH} and N_{FH} as population of self-trapped and free hole, respectively; for electron, N_{TE} and N_{FE} as population of electron bound to STH and free electron, respectively; k_{HT} as hole intrinsic trapping rate constant and k_{ET} as electron capture rate constant by STH. Then we have following rate equations

$$\frac{dN_{TH}}{dt} = N_{FH} k_{HT} \qquad (4)$$

$$\frac{dN_{FH}}{dt} = N_0 - N_{FH} k_{HT} \qquad (5)$$

$$\frac{dN_{TE}}{dt} = k_{ET} N_{FE} (N_{TH} - N_{TE}) \qquad (6)$$

$$\frac{dN_{FE}}{dt} = N_0 - k_{ET} N_{FE} (N_{TH} - N_{TE}) \qquad (7)$$

where N_0 is initial photoexcited carrier density.

The transient absorption signal of free carrier contains contribution from both electron and hole as $TA \propto \alpha N_{FH} + (1 - a) N_{FE}$ and their relative contribution α is inversely proportional to their effective masses. For Sb_2S_3, hole effect mass is generally larger than electron thus α<0.5. The free carrier decay kinetics at different densities (Fig. 4b in main text) can be best replicated with α ~ 0.2, k_{HT} ~ 0.6 ps^{-1} and k_{ET} ~ 6×10^{-21} cm^{-3} ps^{-1}.

Supplementary References

1. Bube RH. Trap Density Determination by Space‐Charge‐Limited Currents. *J. Appl. Phys.* **33**, 1733-1737 (1962).

2. Adinolfi V, *et al.* The In-Gap Electronic State Spectrum of Methylammonium Lead Iodide Single-Crystal Perovskites. *Adv. Mat.* **28**, 3406-3410 (2016).

3. de Jong M, Seijo L, Meijerink A, Rabouw FT. Resolving the ambiguity in the relation between Stokes shift and Huang–Rhys parameter. *Physical Chemistry Chemical Physics* **17**, 16959-16969 (2015).

4. Türck V, *et al.* Effect of random field fluctuations on excitonic transitions of individual CdSe quantum dots. *Phys. Rev. B* **61**, 9944-9947 (2000).

5. Zhao H, Kalt H. Energy-dependent Huang-Rhys factor of free excitons. *Phys. Rev. B* **68**, 125309 (2003).

6. Lao X, *et al.* Luminescence and thermal behaviors of free and trapped excitons in cesium lead halide perovskite nanosheets. *Nanoscale* **10**, 9949-9956 (2018).

7. Luo J, *et al.* Efficient and stable emission of warm-white light from lead-free halide double perovskites. *Nature* **563**, 541-545 (2018).

8. Lemos AM, Markham JJ. Calculation of the Huang-Rhys factor for F-centers. *Journal of Physics and Chemistry of Solids* **26**, 1837-1851 (1965).

9. Lambkin JD, Dunstan DJ, Homewood KP, Howard LK, Emeny MT. Thermal quenching of the photoluminescence of InGaAs/GaAs and InGaAs/AlGaAs strained‐layer quantum wells. *Appl. Phys. Lett.* **57**, 1986-1988 (1990).

10. Bacher G, *et al.* Influence of barrier height on carrier dynamics in strained InxGa1−xAs/GaAs quantum wells. *Phys. Rev. B* **43**, 9312-9315 (1991).

11. Martin P, *et al.* Subpicosecond study of carrier trapping dynamics in wide-band-gap crystals. *Phys. Rev. B* **55**, 5799-5810 (1997).
